# Growth performance, nutrient digestibility, antioxidant state, ileal histomorphometry, and cecal ecology of broilers fed on fermented canola meal with and without exogenous enzymes

**DOI:** 10.1007/s11250-023-03476-9

**Published:** 2023-01-26

**Authors:** Ahmed M. Elbaz, Said E. El-sheikh, Ahmed Abdel‑Maksoud

**Affiliations:** Desert Research Center, Mataria, Cairo Egypt

**Keywords:** Broilers, Fermentation, Exogenous enzymes, Performance, Digestive, Antioxidant state

## Abstract

This study was conducted to evaluate the effects of supplementation of exogenous enzymes in broiler diets that includes fermented canola meal on performance, nutrient digestibility, biochemical indication, antioxidative capacity, digestive enzyme activity, immune responses, and gut health. Five hundred 1-day-old Ross 308 broiler chicks were randomly allocated into five experimental groups (5 replicate/group), the first group: a control (CON) contained a basal diet, and the second to the fifth groups were fed diets as follows: containing 20% canola meal (CM), contains 20% fermented canola meal (FCM), contains 20% canola meal and exogenous enzymes at 0.02%/kg feed (ECM), and contains 20% fermented canola meal and exogenous enzymes at 0.02%/kg feed (EFC), respectively. At the finisher phase, the best body weight gain, feed conversion ratio, and nutrient utilization were associated with chickens fed EFC compared to other groups (*P* < 0.05). Total protein, albumin, alanine aminotransferase, and superoxide dismutase levels increased (*P* < 0.05), while cholesterol and malondialdehyde levels decreased in chickens fed on EFC. Likewise, there was a significant increase in the relative weight of the bursa of Fabricius and antibody titer against Newcastle disease, whereas the weight of abdominal fat decreased in the EFC group compared to other groups. Furthermore, there was a significant improvement in the activity of lipase and amylase enzymes (*P* < 0.05) in the EFC group. Fermented canola meal addition improved gut health (decreased *Escherichia coli*, increased *Lactobacillus*, and the highest values of villus height). Overall, these results confirmed that supplementing a fermented canola meal diet with exogenous enzymes improved growth performance through enhancing nutrient digestibility, immunity, antioxidant capacity, and gut health. Thus, adding enzymes to a diet containing fermented canola meal can be recommended as an alternative protein source that could be safely used to replace up to 20% soybean meal in broiler diets.

## Introduction

In poultry production systems, dietary costs and diseases are the most important economic challenges (Jayaraman et al. 2013). The continuous increase in feed ingredient prices globally, especially corn and soybean meal, is linked to an increase in demand from developing countries. In Egypt, about 85–90% of the food raw materials are imported, which constitutes an increase in the costs of the poultry industry. Therefore, nutritionists began to search for alternatives such as oil extraction waste to use in poultry feed to reduce feeds cost. However, it should be taken into consideration the “anti nutritional” that affects the utilization of oil extraction waste (Min et al. 2009).

Canola meal (CM) is a good protein to be used to replace parts of soybean meal in the poultry diet for its higher crude protein content of approximately 35–40% and its higher contents of sulfur-containing amino acids (Newkirk 2009). However, it contains some anti-nutritional factors (glucosinolates, tannins, crude fiber, and phytate) that impact the utilization of broiler feed (Kocher et al. 2000). There are many methods that help to reduce anti-nutritional factors. One of these methods is fermentation. Some studies have shown that adding probiotics or using the fermentation process for some dietary ingredients has improved performance, increased nutrient digestibility, and reduced effects of anti-nutrition (Chiou et al. 2000; Elbaz et al. 2021). Furthermore, the fermentation process leads to enhanced gut health by reducing pathogens such as *E. coli*, and *Clostridium perfringens* which resulted in reduced use of antibiotics as a drug or growth promoter (Elbaz 2021; Feng et al. 2007).

Although many reports indicated that the nutritional value of the feed ingredient improved through the fermentation process (Elbaz 2021; Li et al. 2020), we did not reach the desired results, so we used exogenous enzymes to enhance the nutritional value of the canola meal in this study. Especially with the monogastric animal’s inability to digest fibers. Therefore, the addition of exogenous enzymes is important to degrade complex fibers (non-starch polysaccharides, NSP) to improve the nutritional value of unconventional feed ingredients (Jiang et al. 2014; Cowieson 2010). High levels of NSP in the diet lead to the binding of a large amount of water, which causes the fluid viscosity increases which may cause some problems in the digestion of dietary (carbohydrate, protein, and fat) and therefore reduce the utilization of nutrients (Alagawany et al. 2018). Enzymes play an influential role in improving feed digestion and utilization (Cowieson et al. 2006). Some reports also reported that the supplementation of enzymes increased the solubility of NSP, which improved the utilization of energy and protein (Olukosi et al. 2015). Furthermore, exogenous enzymes improved nutrient digestibility in poultry leading to lowering nutrient excretion in excreta such as excess nitrogen, phosphorus, copper, and zinc (Berwanger et al. 2017; Moghaddam et al. 2012) which reduces environmental pollution and improves feed utilization. Several studies indicated that supplementing broiler feed with exogenous enzymes reduced the effect of anti-nutrients and improved productive performance (Cowieson et al. 2006; Baidoo et al. 2003). Therefore, we presumed that the addition of exogenous enzymes with fermented canola will help reduce anti-nutritional factors, thus increasing the nutritional value and improving performance and gut health. The current study was conducted to evaluate the growth performance, and digestibility, to disclose serum biochemical variables, antioxidant status, digestive enzymes activity, immune responses, intestinal morphology, and microflora in broilers fed with diets including fermented canola meal and exogenous enzymes to explore the possibilities of replacing soybean meal with canola meal in broiler diets.

## Materials and methods

### Experimental design and birds

Broilers (Ross 308) were obtained as day-old chicks (total of 500 chicks), weighed, and randomly assigned to five treatments (100 chicks/group). Each treatment contained five replicates (20 chicks/ replicate). The five treatment diets were as follows: CON; 0% CM diet as basal diet, CM; 20% CM diet as control, FCM; 20% fermented CM diet, ECM; 20% CM diet with no multi-enzyme added (0.02%/kg), and EFC; 20% fermented CM diet with multi-enzyme added. The enzyme complex used in this study was Allzyme® SSF, Alltech, which contains protease, amylase, β-glucanase, xylanase, pectinase, cellulase, and phytase. The diet (Table 1) was prepared according to the needs of the bird on a foundation NRC (1994). The water and mashed diets ad libitum were available throughout the 42-day trial period. The chicks were subjected to 24 h of light for the first week, and then daylight hours were reduced to 22 h per day till the end of the experiment. The room temperature was maintained at 32.0 ± 0.5 °C during the first 3 days, then gradually reduced to 28 °C by the end of week 2, and gradually reduced to 21 °C by the end of the experiment. On day 7, NDV plus and avian influenza (H5N1) vaccines were injected subcutaneously in the back of the neck and on days 17 and 27 were inoculated against Newcastle disease virus (NDV) in the water.

**Table 1:** Ingredient and chemical composition of diets used for feeding broiler

	Starter diet					Finisher diet				
	CON	CM	FCM	ECM	EFC	CON	CM	FCM	ECM	EFC
Ingredient										
Canola meal	-	20	-	20	-	-	20	-	20	-
Fermented Canola meal	-	-	20	-	20	-	-	20	-	20
Yellow corn	53.50	50.08	50.08	50.08	50.08	63.50	57.21	57.21	57.21	57.21
Soybean meal (48%)	34.38	15.17	15.17	15.17	15.15	27.20	11.49	11.49	11.49	11.49
Corn gluten	5.32	7.70	7.70	7.70	7.70	3.00	3.34	3.34	3.34	3.34
Corn oil	2.50	2.95	2.95	2.95	2.95	2.52	4.50	4.50	4.50	4.50
Di calcium phosphate	2.00	1.95	1.95	1.95	1.95	1.75	1.66	1.66	1.66	1.66
Limestone	1.40	1.17	1.17	1.17	1.17	1.15	0.92	0.92	0.92	0.92
Premix*	0.30	0.30	0.30	0.30	0.30	0.20	0.20	0.20	0.20	0.20
Salt	0.30	0.30	0.30	0.30	0.30	0.20	0.20	0.20	0.20	0.20
DL-methionine	0.13	0.12	0.12	0.12	0.12	0.20	0.18	0.18	0.18	0.18
L-lysine	0.17	0.26	0.26	0.26	0.26	0.28	0.30	0.30	0.30	0.30
Multi-enzyme**	-	-	-	0.02	0.02	-	-	-	0.02	0.02
Chemical composition										
ME (kcal/kg)	3000	3000	3000	3000	3000	3150	3150	3150	3150	3150
Crude protein	23	23	23	23	23	20	20	20	20	20
Calcium	1.08	1.08	1.08	1.08	1.08	0.9	0.9	0.9	0.9	0.9
Available phosphorus	0.52	0.52	0.52	0.52	0.52	0.45	0.45	0.45	0.45	0.45

^*^Vitamin A 12,000 IU, vitamin B12 0.02 mg, vitamin D3 3000 IU, vitamin E 40 mg, vitamin K3 3 mg, vitamin B2 6 mg, vitamin B1 2 mg, vitamin B6 5 mg, biotin 0.075 mg, niacin 45 mg, folic acid 2 mg, pantothenic acid 12 mg, manganese 100 mg, zinc 600 mg, iron 30 mg, selenium 0.2 mg, copper 10 mg, iodine 1 mg, cobalt 0.1 mg. **Protease, pectinase, amylase, β-glucanase, cellulase, xylanase, and phytase

### Fermentation process

Canola meal was obtained from the Environmental Section of the Desert Research Center. The meal was separately inoculated which each of the *Lactobacillus fermentum* and *Bacillus subtilis* at 0.1% based on the dry matter of the canola meal. The probiotic strains were mixed with distilled water to prepare a solution for spraying dry canola to begin the fermentation process (to elevate moisture to 70%) and were mixed thoroughly manually (Pandey et al. 2008). The treated canola was spread on the floor of a well-ventilated room, keeping the room temperature at 30–32 °C for 1 week with continuous stirring. After the fermentation period, fermented CM was dried at 50 °C for 2 days and then packed in bags, until mixed with the experiment diet.

### Performance and carcass traits

Feed intake (FI) and live body weight (LBW) were weekly recorded manually. Body weight gain (BWG) and feed conversion ratio (FCR) values were calculated. Mortality was also recorded daily for each group individually. At the age of 42 days, ten chickens were slaughtered from each treatment to estimate the specifications of the carcass such as dressing, carcass yield, and abdominal fat. Lymphoid organs such as the thymus, spleen, and bursa of Fabricius were weighed as an immune index.

### Blood biochemistry

On day 42, before slaughter (10 birds/ group), blood samples were drawn from the jugular vein and then centrifuged (3000 rpm for 15 min) to obtain the serum. Total protein, albumin, creatinine, uric acid, triglycerides, total cholesterol, glucose, aspartate aminotransferase (AST), and alanine aminotransferase (ALT) were estimated by a spectrophotometer (Milton Roy, Ivyland, PA, USA) according to the manufacturer’s instructions (Spinreact Co., Girona, Spain). Assays of superoxide dismutase (SOD), glutathione peroxidase (GSH-Px), and malondialdehyde (MDA) were examined using commercial kits (Spinreact Co. Girona, Spain) as an indicator of antioxidant capacity. To detect antibody titer against (NDV) and avian influenza virus (AIV) in serum samples, the hemagglutination inhibition (HI) test was used according to El-Moneim, et al. (2020) and Saleh et al. (2020) respectively.

### Digestibility and digestive enzymes activity

On day 42, ten broilers (2 birds/ replicated) per group were taken and placed in battery cages with a wire mesh bottom and excreta collection trays. Each cage has separate water and feed sources for each bird. The birds were starved for 12 h before the start of the digestion experiment (to initialize). Thereafter, excreta were collected for 3 days, with an average of every 8 h each day. The remaining feed and feathers in the excreta trays were carefully removed and weighed. Excreta were dried and stored in sealed bags at − 10 °C for analysis later. Feed and excreta samples were analyzed for dry matter (DM), crude protein (CP), ether extract (EE), and nitrogen-free extract using routine procedures (AOAC 1990). During slaughter at 42 days, samples of intestine contents (5 birds/ group) were collected and placed in a neutral saline solution for preservation. At the start of the analysis, the solution was separated through centrifugation (1792 g for 15 min) and then separated the supernatant part to estimate the activity of digestive enzymes. Lipase (Sklan and Halevy 1985), amylase (Pinchasov et al. 1990), and trypsin (Sklan et al. 1975) were evaluated.

### Histological and microflora enumeration

The samples of ileum were collected (5 birds/group) as segments of approximately 2 cm taken from the mid-point. Segments were fixed in a 10% neutral-buffered formalin solution and embedded in paraffin wax. Then stained with hematoxylin and eosin, and then the slides were examined under the electron light microscope (ZEISS Axio Imager. A2) to measure histological evidence of ilea such as villus height (VH) and crypt depth (CD) and calculated the villus height/crypt depth ratio (VH/CD).

Samples were taken from the cecum directly after slaughter (five per group), about 5 g, and placed in a homogeneous solution and a serial of decimal dilution was prepared. An agar medium for enumeration was prepared for the targeted bacteria in this experiment (using conventional microbiological techniques). In particular, total *Lactobacillus*, total coliform, and *Escherichia coli* were enumerated using nutrient agar according to Czerwiński et al. (2012). Results were estimated as log10 colony-forming units per gram of cecal digesta.

### Statistical analysis

All data collected were subjected to analysis using the one-way ANOVA procedure of SPSS (SPSS version 22). The effect of treatment difference will be compared using Duncan’s multiple area tests (*P* < 0.05).

## Results

### Performance and carcass measurements

The obtained results showed that adding exogenous enzymes to diets containing fermented canola meal significantly improved the productive performance, as shown in Table 2. BWG and FCR were significantly lower in the group fed on an untreated canola meal diet compared with the control group. There was a noticeable improvement (*P* < 0.05) in BWG and FCR in chickens fed a fermented canola meal diet supplemented with enzymes (EFC) during all experimental stages. However, the FI differed during the experimental stages, as it was not affected (*P* < 0.05) during the starter stage (0–21 days), while it was affected during the other stages (22–42 days and 0–42 days). Chickens fed with EFC showed the best BWG and FCR compared to the rest of the experimental treatments. Interestingly, chickens with fed EFC diets had a higher FI (*P* < 0.05) than the other groups; however, it recorded the best FCR (*P* < 0.05). Furthermore, the percentage of mortality decreased in the groups fed on FCM, ECM, and EFC (*P* < 0.05); compared to those groups fed on CM, the best was the FCM group. The effect of treated canola meal on the carcass traits is shown in Table 2. At 42 days, EFC and FCM groups recorded the lowest relative weight of abdominal fat (*P* < 0.05) compared to CM and other groups. Relative weights of dressing, carcass yield, and liver were not affected by the different dietary treatments (*P* < 0.05).

**Table 2:** Performance of broilers fed different dietary treatments at 42 days

	CON	CM	FCM	ECM	EFC	SEM	P-value
0–21d							
FI (g/b)	942	933	938	941	944	7.220	0.815
BWG (g/b)	743^a^	720^b^	741^a^	737^a^	745^a^	24.61	0.030
FCR	1.259^b^	1.295^a^	1.266^b^	1.276^ab^	1.267^b^	0.085	0.024
22–42d							
FI (g/b)	1929^a^	1754^b^	1920^a^	1885^ab^	1918^a^	34.80	0.029
BWG (g/b)	1032^a^	875^b^	1020^a^	997^ab^	1063^a^	71.00	0.016
FCR	1.868^b^	2.004^a^	1.882^b^	1.891^b^	1.805^c^	0.121	0.001
0–42d							
FI (g/b)	2871^a^	2687^b^	2859^a^	2826^a^	2861^a^	42.55	0.042
BWG (g/b)	1776^ab^	1595^c^	1761^ab^	1732^b^	1808^a^	39.10	0.001
FCR	1.617^b^	1.686^a^	1.623^b^	1.632^b^	1.582^c^	0.107	0.011
Mor. (%)	2	4	0	2	2	-	-
Carcass traits (%)							
Dressing	70.30	69.85	70.11	70.8	71.05	3.251	0.203
Carcass yield	75.23	74.09	74.50	75.14	74.61	1.545	0.171
Liver	2.18	2.05	2.23	2.21	2.35	0.252	0.610
Abdominal fat	1.70^a^	1.58^ab^	1.34^b^	1.68^a^	1.39^b^	0.430	0.001

^a^^,b,c^Each trait with different superscripts differ significantly at *p* < 0.05; *FI*, feed intake; *BWG*, body weight gain; *FCR*, feed conversion ratio; *Mor.*, mortality; *CON*, basal diet as control group; *CM*, added 20% CM in diet no processing; *FCM*, added 20% fermented CM in diet; *ECM*, added 20% CM plus multi-enzyme in diet; and *EFC*, added 20% FCM in diet with multi-enzyme

### Digestibility and digestive enzymes

Table 3 shows the impact of feeding a treated canola meal on the digestibility of nutrients in chickens. Digestibility of DM, CP, and EE in chicks fed with FCM, EFC, and ECM diet increased (*P* < 0.05) compared to those fed with CM, while the results are similar to chicks fed with the control diet (CON). Interestingly, broilers fed with EFC and FCM had the best digestion of DM, CP, and EE. However, there was no difference in nitrogen-free extract utilization (*P* < 0.05).

**Table 3:** Nutrient digestibility (%) and digestive enzymes activities (U/ml) of broilers fed different dietary treatments at 42 days

	CON	CM	FCM	ECM	EFC	SEM	*P*-value
Nutrient digestibility							
Dry matter	78.1^a^	74.2^b^	78.4^a^	76.4^ab^	77.9^a^	1.073	0.020
Crude protein	87.4^a^	80.0^b^	86.8^a^	84.2^ab^	88.2^a^	0.541	0.016
Ether extract	65.0^bc^	60.8^c^	69.3^a^	68.7^a^	70.2^a^	1.760	0.032
Nitrogen-free extract	81.5	80.4	82.0	81.1	81.7	0.475	0.181
Digestive enzymes activities							
Lipase	8.41b	8.22b	11.7a	9.35ab	10.9a	12.25	0.003
Amylase	5.06c	4.84c	5.78b	6.37a	5.95b	8.461	0.011
Trypsin	22.5	21.8	22.3	21.6	22.1	21.37	0.227

^a,b,c^Each trait with different superscripts differ significantly at *p* < 0.05; *CON*, basal diet as control group; *CM*, added 20% CM in diet no processing; *FCM*, added 20% fermented CM in diet; *ECM*, added 20% CM plus multi-enzyme in diet and EFC added 20% FCM in diet with multi-enzyme

Digestive enzyme activities (lipase, amylase, and trypsin) in the intestine are shown in Table 3. The highest activity of lipase enzyme (*P* < 0.05) was recorded in birds fed on fermented canola meal (FCM and EFC). On the other hand, there was a noticeable increase in the activity of amylase enzyme (*P* < 0.05) in birds fed with only exogenous enzymes (ECM) compared to the other groups. The activity of trypsin was not affected by the experimental treatments.

### Immune response

The immune response of broilers fed with fermented canola meal with or without exogenous enzymes was evaluated by the relative weight of lymphoid organs and humoral immune response, as shown in Table 4. The weight of the thymus and spleen and AIV levels was not affected by any dietary experiment. However, a significant difference (*P* < 0.05) was observed in the weight of the bursa of Fabricius; among experimental treatments, the highest values (*P* < 0.05) were observed in birds fed FCM and EFC in a comparison with other groups. Likewise, the highest (*P* < 0.05) antibody levels against NDV were in birds fed FCM and EFC compared to the values in other groups.

**Table 4:** Lymphoid organs and humoral immune response of broilers fed different dietary treatments at 42 days

	CON	CM	FCM	ECM	EFC	SEM	*P*-value
Thymus	0.26	0.21	0.19	0.21	0.23	0.182	0.160
Bursa of fabricius	0.076^ab^	0.059^b^	0.089^a^	0.62^b^	0.083^a^	0.027	0.043
Spleen	0.23	0.21	0.19	0.20	0.22	0.180	0.110
NDV	5.57^b^	5.05^b^	6.88^a^	5.41^b^	6.76^a^	0.224	0.002
AIV	3.44	3.19	3.73	3.56	4.17	0.157	0.091

^a,b,c^Each trait with different superscripts differ significantly at *p* < 0.05; *CON*, basal diet as control group; *CM*, added 20% CM in diet no processing; *FCM*, added 20% fermented CM in diet; *ECM*, added 20% CM plus multi-enzyme in diet; and *EFC*, added 20% FCM in diet with multi-enzyme. *NDV*, Newcastle disease virus; *AIV*, avian influenza virus (H9N1)

### Serum biochemical and antioxidant status

Table 5 shows, serum total protein, and albumin concentrations were significantly (*P* < 0.05) elevated in FCM and EFC groups, while cholesterol concentrations decreased (*P* < 0.05) in FCM, ECM, and EFC groups compared with CM. However, no differences were noticed between experimental groups in glucose, triglycerides, uric acid, and creatinine at 42 days. Furthermore, AST enzyme levels increased in ECM and EFC groups compared to the CM and other groups (*P* < 0.05). However, the level of the enzyme ALT was not affected by dietary treatments. The antioxidant status of birds fed on fermented canola meal only or mixed with multienzyme was evaluated by estimating blood SOD, MDA, and GPx, as shown in Table 6. Broilers fed with a diet including FCM, ECM, and EFC exhibited higher (*P* < 0.05) levels of SOD and lower (*P* < 0.05) levels of MDA compared to those fed the other diets. Birds fed on EFC had higher SOD and lower MDA. However, the level of GSH-Px was not influenced by dietary treatments (*P* < 0.05).

**Table 5:** Serum biochemical of broilers fed different dietary treatments at 42 days

	CON	CM	FCM	ECM	EFC	SEM	*P*-value
Total protein (g/dL)	5.51^b^	5.46^b^	5.98^ab^	6.05^ab^	6.28^a^	0.441	0.016
Albumin (g/dL)	3.47^a^	3.12^b^	3.50^a^	3.37^a^	3.41^a^	1.645	0.037
uric acid (mmol/L)	133	135	131	136	132	0.134	0.210
Creatinine (mmol/L)	17.3	17.1	16.9	17.5	17.3	0.221	0.130
Triglycerides (mg/dL)	167	171	162	173	159	6.541	0.296
Cholesterol (mmol/L)	4.24^a^	4.17^a^	3.88^ab^	3.35^b^	3.72^ab^	2.450	0.030
Glucose (mmol/L)	6.26	5.95	6.47	6.87	6.05	0.596	0.701
ALT (U/L)	5.26	5.51	5.11	5.37	5.29	0.388	0.123
AST (U/L)	237^b^	246^b^	261^ab^	278^a^	265^ab^	16.43	0.011

^a,b,c^Each trait with different superscripts differ significantly at *p* < 0.05; *CON*, basal diet as control group; *CM*, added 20% CM in diet no processing; *FCM*, added 20% fermented CM in diet; *ECM*, added 20% CM plus multi-enzyme in diet; and EFC, added 20% FCM in diet with multi-enzyme. *LDL*, low-density lipoprotein; *HDL*, high-density lipoprotein; *AST*, aspartate aminotransferase; *ALT*, alanine aminotransferase

**Table 6:** Antioxidant capacity of broilers fed different dietary treatments at 42 days

	CON	CM	FCM	ECM	EFC	SEM	*P*-value
SOD (U/mL)	7.26^ab^	6.55^b^	8.14^a^	7.09^ab^	7.81^a^	1.271	0.036
MDA (nmol/L)	3.01^b^	3.49^a^	3.11^b^	3.57^a^	3.05^b^	0.920	0.015
GSH-Px (U/mL)	186.2	180.0	189.5	178.9	184.8	2.335	0.127

^a,b,c^Each trait with different superscripts differ significantly at *p* < 0.05; *CON*, basal diet as control group; *CM*, added 20% CM in diet no processing; *FCM*, added 20% fermented CM in diet; *ECM*, added 20% CM plus multi-enzyme in diet; and *EFC*, added 20% FCM in diet with multi-enzyme. Superoxide dismutase (SOD); methane dicarboxylic aldehyde (MDA); glutathione peroxidase (GSH-Px)

### Microbial enumeration and histomorphology

Screening of cecal microbial enumeration (*Lactobacillus, Escherichia coli,* and *Total Coliform*) of broilers fed with different dietary treatments is presented in Table 7. The *E. coli* population significantly decreased (*P* < 0.05) in cecal contents due to feeding on the FCM and EFC compared to the other group. Moreover, the *Lactobacillus* population increased significantly in the birds fed FCM and EFC groups compared to the birds fed the rest of the treated groups. While, the count of *Total Coliform* in cecal contents was not affected by the experimental dietary (*P* < 0.05). Results in Table 7 revealed that feeding on a diet containing FCM and EFC exhibited significant enhancement (*P* < 0.05) in villus height (VH) compared to the rest groups (*P* < 0.05), even though the crypt depth (CD) and VH/CD ratio were not significantly (*P* < 0.05) different throughout experimental groups.

**Table 7:** Histological and microbial counts (log10 CFU g^−1^) of broilers fed different dietary treatments at 42 days

	CON	CM	FCM	ECM	EFC	SEM	*P*-value
Histological							
Villus height (μm)	489^b^	456^b^	556^a^	465^b^	537^a^	8.250	0.001
Crypt depth (μm)	110	98	112	105	108	2.541	0.130
VH/CD ratio	4.45	4.64	4.97	4.43	4.96	0.107	0.071
Microbial count							
* Lactobacilli*	6.93^b^	6.80^b^	7.75^a^	6.76^b^	7.60^a^	0.065	0.010
* Escherichia coli*	7.59^ab^	8.16^a^	7.12^b^	7.95^a^	7.34^b^	0.070	0.026
Total coliform	5.16	4.90	5.06	5.11	5.09	0.109	0.094

^a,b,c^Each trait with different superscripts differ significantly at *p* < 0.05; *CON*, basal diet as control group; *CM*, added 20% CM in diet no processing; *FCM*, added 20% fermented CM in diet; *ECM*, added 20% CM plus multi-enzyme in diet; and *EFC*, added 20% FCM in diet with multi-enzyme. *VH/CD*, villus height/crypt depth ratio

## Discussion

A previous study showed that the fermentation process improved the nutritional value of the canola meals and enhanced growth performance, even though we did not obtain the desired results (Elbaz 2021). Therefore, it was speculated that adding exogenous enzymes might enhance the nutritional value of fermented canola meals, which should reflect on improving the productive performance with the potential of replacing soybean meal in larger quantities in a broiler diet. In this study, we found that adding exogenous enzymes changed the nutritional characteristics of the fermented canola meal. Our results showed a significant improvement in growth performance in chickens fed on EFC and FCM diets, which is indicated by the increase in BWG and the decrease in FCR during all the experimental stages compared with other groups. Interestingly, chickens fed with EFC had the highest BWG and lowest FCR (*P* < 0.05). The improvement in growth performance in the group fed on fermented canola meal with exogenous enzymes may be due to the enhancement in nutritional values, the increase in digestibility, and the palatability of the feed ingredients as a result of the fermentation process with the addition of some enzymes. The enhanced FCR values seen in all treatments during the experiment could be due to the increase in FI for the birds to meet the needs of growth requirements. Several studies reported an improvement in BWG and FCR due to the fermentation process of unconventional feed ingredients or supplementing it with exogenous enzymes (Chiang et al. 2009; Xu et al. 2012). Similar to the results of this study, previous studies reported that the fermentation process or adding exogenous enzymes improved nutritional values (Elbaz 2021; Skrede et al. 2003) through the degradation of fibers; the breakdown of protein (more amino acids available), non-starch polysaccharides, also, led to a reduction in antinutritional content which increases the nutritional value of unconventional feed ingredients (Jakobsen et al. 2015). It also showed that crude protein content increased in the dry matter in the diet as a result of the fermentation process (Murekatete et al. 2012). Furthermore, adding exogenous enzymes as a supplement to the diet enhanced the broiler chicken’s digestibility by increasing substrate availability, which led to accelerating the activity of digestive enzymes and consequently enhanced the building units of protein, lipids, and carbohydrates of intestinal transporters by increasing nutrient transport capacity through the intestinal epithelial cells (Horvatovic et al. 2015). These different effects of the fermentation process and the addition of enzymes to canola meal explain the reason for the improvement in the general performance of the bird and the enhanced digestion. Therefore, the noticeable improvement in the growth performance of chickens fed on EFC can be explained by the synergistic positive effect of adding enzymes to the fermented canola meal, which enhances the nutritional value and increases the available nutrients, in addition to the improvement of the intestinal composition (histological and microbial), as our results show later. The results of the current study showed a significant decrease in the relative weight of abdominal fat in chickens fed with FEC and FCM in comparison with the other experimental groups, while the rest of the carcass characteristics were not affected. These results are in agreement with those obtained by Bidura et al. (2007), who noticed that the abdominal fat content in broiler chickens fed a diet that included probiotics was significantly lower compared with the control group. The decreased relative weight of abdominal fat in the groups fed on fermented canola can be explained by the role of beneficial microbes (probiotics) in the conversion of excess energy from the metabolism process or by the rate of fatty acid synthesis being reduced through a decreased in the activity of acetyl-CoA carboxylase (responsible enzyme limiting in fatty acid synthesis), by Santoso et al. (1995).

The present study results showed that adding exogenous enzymes to the fermented canola meal improved the nutritional value by enhancing DM, CP, and EE. Looking at the previous results, one of the most important strategies that are used to improve the nutritional value of unconventional feed ingredients in chicken diets is the use of some biological additives or treatments, such as the fermentation process and the addition of enzymes, where previous experiments indicated that the fermentation process or the addition of enzymes led to a lowering the fiber content (Shahowna et al. 2013), improved protein solubility (Nie et al. 2015), increased lipid content, increased crude protein content, improved vitamin availability (Canibe and Jensen 2012; Elbaz 2021), and increased feedstuff palatability (Shahowna et al. 2013). Furthermore, reduced antinutritional factors such as glucosinolate, phytate, and tannins (Chiang et al. 2009; Sokrab et al. 2014). The current study clarified the significant impact of experimental dietary treatments on the activity of digestive enzymes. The activity of both amylase and lipase enzymes increased in broilers fed with a ration containing fermented canola or exogenous enzymes. Following up on the results of previous studies, it was clear that adding exogenous enzymes or fermenting raw feed materials is used in poultry feed to improve the nutritive value of the feed by facilitating the breakdown of antinutritional (such as phytic acid and dietary fiber) and increasing the activity of digestive enzymes (Hamdi et al. 2018) that lead to enhancing feed efficiency and improve productive performance of the chickens (Zou et al. 2013). That data may explain our results about the performance improvement (enzyme activities) of broiler chicken fed fermented canola meal or exogenous enzymes. These findings reflect how fermenting the canola meal and adding exogenous enzymes enhanced metabolism and absorption for broilers.

The results of the current study showed a reduction in total cholesterol concentrations in broilers fed on EFC and FCM compared to other groups. Similar hypocholesterolemic effects of probiotics added on serum lipids of chickens had been reported by Mayahi et al. (2009). The decrease in the cholesterol level in the blood can be explained by the fact that some strains of probiotics may contain bile salt hydrolase (BSH) activity, which led to the deconjugation of bile salts (Klaver and van der Meer 1993). Moreover, it may be that some lactic acid bacteria can assimilate cholesterol into their cells, which led to cholesterol reduction in the surrounding environment (Gilliland et al. 1985). In addition, this study’s results showed including fermented canola meal or adding enzymes to the diet significantly increased the level of total protein and albumin in the blood. Similar observations have also been made in chickens receiving feed with FSBM (Feng et al. 2007). The diet with fermented canola meal or exogenous enzymes also caused an increase in AST activity. A similar effect was reported by Otto-Ślusarczyk et al. (2016). Possibly, the cause of increased AST enzyme concentrations is the result of an increase in the rate of amino acid transformation, especially with the increase in protein metabolism, and no symptoms appeared with diagnostics of liver disease.

Oxidative stress was studied because of its severe damage to the cells and the metabolism process, which damages protein, nucleic acids, and some biological macromolecules (produce large amounts of MDA), which damage tissues and thus make them more susceptible to diseases. Our results indicate that feeding broiler chickens a diet that includes fermented canola meal with the supplementation of exogenous enzymes improved antioxidant status by significantly increasing the level of SOD and decreasing MDA (*P* < 0.05). In agreement with our results, Drażbo et al. (2018) found that the fermentation of rapeseed cake could be beneficial in oxidation resistance and promoting antioxidant capability. These results confirm that the cells were not under stress which is confirmed by the reduced level of MDA. The reason for the low level of MDA can be due to the increase in free amino acids, such as tyrosine, methionine, and lysine as a result of the fermentation process, which plays a role as an antioxidant (Wang and De Mejia 2005). Another explanation, due to the action of microbial glucosidases, some lipophilic aglycones are produced during the fermentation process, which are more effective scavengers of free radicals than the corresponding glycosides (Lin et al. 2006) leading to an increase in oxidative activity. In numerous researches, dietary probiotics have been proven to be beneficial in promoting the activities of antioxidant enzymes and reducing the adverse influence of oxidative stress (Deng et al. 2012), thus improving the health of the bird (host).

The results of the current study indicated that adding exogenous enzymes to broiler diets containing fermented canola (EFC) had a beneficial effect on antibody production against Newcastle disease compared to the other experimental groups (*P* < 0.05). This shows that the manipulation of gut microbiota by feeding on fermented canola meal (as a role probiotic) influences the development of the immune response in birds. These positive effects of improving the immune response to the virus antibodies (by producing a high level of blood antibody) are a result of the fermentation process as one of the ways to use probiotics to improve the health status of the birds, which are in agreement with the findings of Dalloul et al. (2003) and Koenen et al. (2004). Furthermore, the relative weight of the bursa increased in the groups fed on fermented canola meal (EFC and FCM) compared to the rest of the groups. The remarkable improvement in the weight of immune organs (bursa of Fabricius) in chickens fed with fermented canola meal may be due to the improvement in the microbial load inside the intestine, which affects the immune response of broilers. The increase in LAB bacteria in the gut is a result of the fermentation process which had a positive effect on the immune functions of chickens (Missotten et al. 2013); moreover, it may stimulate the immune cells to produce cytokines, which promotes B cell development switching required for antibody production (Lutful Kabir 2009). These results show how birds fed on fermented canola meal with the addition of enzymes had an enhanced immunity, which reflects on the general performance of the birds.

The importance of studying intestinal microbes and mucosa is in their role in host immune defense mechanisms against pathogens, enhancing utilization of nutrition, and gut morphology, which positively influences the general performance of birds. In the present study, the results showed that the fermentation process had a beneficial modulator effect on the cecal microflora in broilers, which appeared in the increase in the populations of *Lactobacillus* (beneficial bacteria), while the population of *E. coli* (harmful bacteria) decreased. The beneficial influences of fermented feeds on broiler health are due to the healthy gut ecosystems, through the high numbers of *Lactobacillus*, low pH, high lactic acid, and acetic acid concentrations (Engberg et al. 2009). In this regard, fermenting certain feed ingredients is an effective strategy in reducing chicken enteric diseases by beneficial modulator effects on the gut microflora (gut health), which is reflected in the reduction of the use of antibiotics and the better performance of birds (Heres et al. 2003).

One of the most important factors that need to be assessed to determine the carrying capacity and health of the intestine is studying villus height (VH), crypt depths (CD), VH:CD ratio, and the maintenance of intestinal mucosal integrity (Alshelmani et al. 2016; Chiang et al. 2009). This study focused on studying villus height (VH), crypt depths (CD), and VH:CD ratio. In the present study, the results showed that the fermenting canola meal had beneficial effects on the gut histomorphology in broilers, meaning that the villus height significantly increased. Similarly, Chiang et al. (2009) and Elbaz (2021) found that feeding fermented feed led to an increase in villus height. The noticeable improvement in intestinal histology may be due to the positive modification of the gut microbiota as a result of the fermented feed by reducing the pathogen such as harmful bacteria, and antinutritional dietary. Furthermore, a study carried out by Montanhini Neto et al. (2013) reported that there was an increase in immunity and activity of the intestinal mucosa intestinal cells when adding multi-enzymes to diets containing unconventional feed ingredients. It may have occurred due to better digestibility of the ingredients and the increase in the breakdown of antinutrients; therefore, the number of nutrients available for growth increases. The current study results showed that fermenting canola meal is more beneficial to broilers on intestinal morphology (Perez-Maldonado et al. 2003). As mentioned previously, adding exogenous enzymes to a broiler diet containing fermented canola meal has multiple positive effects on the nutritional value of feed ingredients: enhancing digestive enzymes activity and immunity, along with improving intestinal mucosa morphology and intestinal ecosystems in broilers, which may be responsible for the growth and feed conversion promotion.

## Conclusion

Adding enzymes to broiler diets containing fermented canola meal (20%) improved nutrient digestibility and digestive enzyme activity and enhanced growth performance, and gut health. Furthermore, it improved antioxidant status, which helps to protect against oxidative stress. Thus, it is concluded that adding enzymes improved the nutritional value of diets that contain fermented canola meal (unconventional feed), which increases the possibility of replacing larger quantities of soybean meal with treated canola meal in broiler feed.

## Data Availability

The datasets generated and analyzed during the current study will be provided upon reasonable request from the corresponding author.

## References

[CR1] Alagawany M, Elnesr SS, Farag MR (2018). The role of exogenous enzymes in promoting growth and improving nutrient digestibility in poultry. Iranian Journal of Veterinary Research.

[CR2] Alshelmani MI, Loh TC, Foo HL, Sazili AQ, Lau WH (2016). Effect of feeding different levels of palm kernel cake fermented by Paenibacillus polymyxa ATCC 842 on nutrient digestibility, intestinal morphology, and gut microflora in broiler chickens. Animal Feed Science and Technology.

[CR3] AOAC, M., 1990. Association of official analytical chemists. Official methods of analysis. AOAC: Off Methods Anal, 1, pp.69–90.

[CR4] Baidoo SK, Yang QM, Walker RD (2003). Effects of phytase on apparent digestibility of organic phosphorus and nutrients in maize–soya bean meal based diets for sows. Animal Feed Science and Technology.

[CR5] Berwanger E, Nunes RV, Pasquetti TJ, Murakami AE, de Oliveira TMM, Bayerle DF, Frank R (2017). Sunflower cake with or without enzymatic complex for broiler chickens feeding. Asian-Australasian Journal of Animal Sciences.

[CR6] Bidura IGNG, Candrawati DPMA, Sumardani NLG (2007). Pengaruh Penggunaan Daun Katuk (Saurupus Androgynus) dan Daun Bawang Putih (Allium Sativum) dalam Ransum terhadap Penampilan Ayam Broiler1. Majalah Ilmiah Peternakan.

[CR7] Canibe N, Jensen BB (2012). Fermented liquid feed—Microbial and nutritional aspects and impact on enteric diseases in pigs. Animal Feed Science and Technology.

[CR8] Chiang G, Lu WQ, Piao XS, Hu JK, Gong LM, Thacker PA (2009). Effects of feeding solid-state fermented rapeseed meal on performance, nutrient digestibility, intestinal ecology and intestinal morphology of broiler chickens. Asian-Australasian Journal of Animal Sciences.

[CR9] Chiou PWS, Chen C, Yu B (2000). Effects of Aspergillus oryzae fermentation extract on in situ degradation of feedstuffs. Asian-Australasian Journal of Animal Sciences.

[CR10] Cowieson AJ (2010). Strategic selection of exogenous enzymes for corn/soy-based poultry diets. The Journal of Poultry Science.

[CR11] Cowieson AJ, Singh DN, Adeola O (2006). Prediction of ingredient quality and the effect of a com h performance and digestible nutrient intake. British Poultry Science.

[CR12] Czerwiński J, Højberg O, Smulikowska S, Engberg RM, Mieczkowska A (2012). Effects of sodium butyrate and salinomycin upon intestinal microbiota, mucosal morphology and performance of broiler chickens. Archives of Animal Nutrition.

[CR13] Dalloul RA, Lillehoj HS, Shellem TA, Doerr JA (2003). Enhanced mucosal immunity against Eimeria acervulina in broilers fed a Lactobacillus-based probiotic. Poultry Science.

[CR14] Deng G, Wang J, Zhang Q, He H, Wu F, Feng T, Zhou J, Zou K, Hattori M (2012). Hepatoprotective effects of phloridzin on hepatic fibrosis induced by carbon tetrachloride against oxidative stress-triggered damage and fibrosis in rats. Biological and Pharmaceutical Bulletin.

[CR15] Drażbo A, Ognik K, Zaworska A, Ferenc K, Jankowski J (2018). The effect of raw and fermented rapeseed cake on the metabolic parameters, immune status, and intestinal morphology of turkeys. Poultry Science.

[CR16] Elbaz AM (2021). Effects of the diet containing fermented canola meal on performance, blood parameters, and gut health of broiler chickens. J. Worlds Poult. Res.

[CR17] Elbaz AM, Ibrahim NS, Shehata AM, Mohamed NG, Abdel-Moneim AME (2021). Impact of multi-strain probiotic, citric acid, garlic powder or their combinations on performance, ileal histomorphometry, microbial enumeration and humoral immunity of broiler chickens. Tropical Animal Health and Production.

[CR18] El-Moneim AEMEA, El-Wardany I, Abu-Taleb AM, Wakwak MM, Ebeid TA, Saleh AA (2020). Assessment of in-ovo administration of Bifidobacterium bifidum and Bifidobacterium longum on performance, ileal histomorphometry, blood hematological, and biochemical parameters of broilers. Probiotics and Antimicrobial Proteins.

[CR19] Engberg RM, Hammershøj M, Johansen NF, Abousekken MS, Steenfeldt S, Jensen BB (2009). Fermented feed for laying hens: effects on egg production, egg quality, plumage condition and composition and activity of the intestinal microflora. British Poultry Science.

[CR20] Feng J, Liu X, Xu ZR, Lu YP, Liu YY (2007). The effect of Aspergillus oryzae fermented soybean meal on growth performance, digestibility of dietary components and activities of intestinal enzymes in weaned piglets. Animal Feed Science and Technology.

[CR21] Gilliland SE, Nelson CR, Maxwell C (1985). Assimilation of cholesterol by Lactobacillus acidophilus. Applied and Environmental Microbiology.

[CR22] Hamdi M, Perez JF, Létourneau-Montminy MP, Franco-Rosselló R, Aligue R, Solà-Oriol D (2018). The effects ofmicrobial phytases and dietary calcium and phosphorus levels on the productive performance and bone mineralization of broilers. Animal Feed Science and Technology.

[CR23] Heres L, Engel B, Van Knapen F, De Jong MC, Wagenaar JA, Urlings HA (2003). Fermented liquid feed reduces susceptibility of broilers for Salmonella enteritidis. Poultry Science.

[CR24] Horvatovic MP, Glamocic D, Zikic D, Hadnadjev TD (2015). Performance and some intestinal functions of broilers fed diets with different inclusion levels of sunflower meal and supplemented or not with enzymes. Brazilian Journal of Poultry Science.

[CR25] Jakobsen GV, Jensen BB, Knudsen KEB, Canibe N (2015). Improving the nutritional value of rapeseed cake and wheat dried distillers grains with solubles by addition of enzymes during liquid fermentation. Animal Feed Science and Technology.

[CR26] Jayaraman S, Thangavel G, Kurian H, Mani R, Mukkalil R, Chirakkal H (2013). *Bacillus subtilis* PB6 improves intestinal health of broiler chickens challenged with Clostridium perfringens-induced necrotic enteritis. Poultry Science.

[CR27] Jiang TT, Feng L, Liu Y, Jiang WD, Jiang J, Li SH, Tang L, Kuang SY, Zhou XQ (2014). Effects of exogenous xylanase supplementation in plant protein-enriched diets on growth performance, intestinal enzyme activities and microflora of juvenile J ian carp (C yprinus carpio var. J ian). Aquaculture Nutrition.

[CR28] Klaver FA, van der Meer R (1993). The assumed assimilation of cholesterol by Lactobacilli and Bifidobacterium bifidum is due to their bile salt-deconjugating activity. Applied and Environmental Microbiology.

[CR29] Kocher A, Choct M, Porter MD, Broz J (2000). The effects of enzyme addition to broiler diets containing high concentrations of canola or sunflower meal. Poultry Science.

[CR30] Koenen ME, Kramer J, Van Der Hulst R, Heres L, Jeurissen SHM, Boersma WJA (2004). Immunomodulation by probiotic lactobacilli in layer-and meat-type chickens. British Poultry Science.

[CR31] Li Y, Guo B, Wu Z, Wang W, Li C, Liu G, Cai H (2020). Effects of fermented soybean meal supplementation on the growth performance and cecal microbiota community of broiler chickens. Animals.

[CR32] Lin CH, Wei YT, Chou CC (2006). Enhanced antioxidative activity of soybean koji prepared with various filamentous fungi. Food Microbiology.

[CR33] Lutful Kabir SM (2009). The role of probiotics in the poultry industry. International Journal of Molecular Sciences.

[CR34] Mayahi, M., Jalali, M.R. and Kiani, R., 2009. Effects of dietary probiotic supplementation on cholesterol and triglyceride levels in broiler chicks' sera. World Poultry Science Association (WPSA), 2nd Mediterranean Summit of WPSA, Antalya, Turkey, pp.4–7.

[CR35] Min YN, Hancock A, Yan F, Lu C, Coto C, Karimi A, Park JH, Liu FZ, Waldroup PW (2009). Use of combinations of canola meal and distillers dried grains with solubles in broiler starter diets. Journal of Applied Poultry Research.

[CR36] Missotten JA, Michiels J, Dierick N, Ovyn A, Akbarian A, De Smet S (2013). Effect of fermented moist feed on performance, gut bacteria and gut histo-morphology in broilers. British Poultry Science.

[CR37] Moghaddam HN, Salari S, Arshami JAVD, Golian A, Maleki MOHSEN (2012). Evaluation of the nutritional value of sunflower meal and its effect on performance, digestive enzyme activity, organ weight, and histological alterations of the intestinal villi of broiler chickens. Journal of Applied Poultry Research.

[CR38] Montanhini Neto R, Ceccantini ML, Fernandes JIM (2013). Immune response of broilers fed conventional and alternative diets containing multi-enzyme complex. Brazilian Journal of Poultry Science.

[CR39] Murekatete N, Hua Y, Kong X, Zhang C (2012). Effects of fermentation on nutritional and functional properties of soybean, maize, and germinated sorghum composite flour. International Journal of Food Engineering.

[CR40] National Research Council, 1994. Nutrient requirements of poultry: 1994. National Academies Press.

[CR41] Newkirk, R., 2009. Canola meal: Feed industry guide. Canadian International Grains Institute, Winnipeg, Canada.

[CR42] Nie C, Zhang W, Ge W, Wang Y, Liu Y, Liu J (2015). Effects of fermented cottonseed meal on the growth performance, apparent digestibility, carcass traits, and meat composition in yellow-feathered broilers. Turkish Journal of Veterinary & Animal Sciences.

[CR43] Olukosi OA, Beeson LA, Englyst K, Romero LF (2015). Effects of exogenous proteases without or with carbohydrases on nutrient digestibility and disappearance of non-starch polysaccharides in broiler chickens. Poultry Science.

[CR44] Otto-Ślusarczyk D, Graboń W, Mielczarek-Puta M (2016). Aspartate aminotransferase–key enzyme in the human systemic metabolism. Postepy Higieny i Medycyny Doswiadczalnej (online).

[CR45] Pandey, A., Soccol, C.R. and Larroche, C. eds., 2008. Current developments in solid-state fermentation. Springer Science & Business Media.

[CR46] Perez-Maldonado RA, Mannion PF, Farrell DJ (2003). Effects of heat treatment on the nutritional value of raw soybean selected for low trypsin inhibitor activity. British Poultry Science.

[CR47] Pinchasov Y, Nir I, Nitsan Z (1990). Metabolic and anatomical adaptations of heavy-bodied chicks to intermittent feeding. 2. pancreatic digestive enzymes. British Poultry Science.

[CR48] Saleh AA, Eltantawy MS, Gawish EM, Younis HH, Amber KA, El-Moneim A, Ebeid TA (2020). Impact of dietary organic mineral supplementation on reproductive performance, egg quality characteristics, lipid oxidation, ovarian follicular development, and immune response in laying hens under high ambient temperature. Biological Trace Element Research.

[CR49] Santoso U, Tanaka K, Ohtani S (1995). Effect of dried *Bacillus subtilis* culture on growth, body composition and hepatic lipogenic enzyme activity in female broiler chicks. British Journal of Nutrition.

[CR50] Shahowna EM, Mahala AG, Mokhtar AM, Amasaib EO, Attaelmnan B (2013). Evaluation of nutritive value of sugar cane bagasse fermented with poultry litter as animal feed. Afr J Food Sci Technol.

[CR51] Sklan D, Halevy O (1985). Digestion and absorption of protein along ovine gastrointestinal tract. Journal of Dairy Science.

[CR52] Sklan D, Hurwitz S, Budowski P, Ascarelli I (1975). Fat digestion and absorption in chicks fed raw or heated soybean meal. The Journal of Nutrition.

[CR53] Skrede G, Herstad O, Sahlstrøm S, Holck A, Slinde E, Skrede A (2003). Effects of lactic acid fermentation on wheat and barley carbohydrate composition and production performance in the chicken. Animal Feed Science and Technology.

[CR54] Sokrab AM, Mohamed Ahmed IA, Babiker EE (2014). Effect of fermentation on antinutrients, and total and extractable minerals of high and low phytate corn genotypes. Journal of Food Science and Technology.

[CR55] Wang W, De Mejia EG (2005). A new frontier in soy bioactive peptides that may prevent age-related chronic diseases. Comprehensive Reviews in Food Science and Food Safety.

[CR56] Xu FZ, Zeng XG, Ding XL (2012). Effects of replacing soybean meal with fermented rapeseed meal on performance, serum biochemical variables and intestinal morphology of broilers. Asian-Australasian Journal of Animal Sciences.

[CR57] Zou J, Zheng P, Zhang K, Ding X, Bai S (2013). Effects of exogenous enzymes and dietary energy on performance and digestive physiology of broilers. Journal of Animal Science and Biotechnology.

